# Expressed Emotion, Shame, and Non-Suicidal Self-Injury

**DOI:** 10.3390/ijerph15050890

**Published:** 2018-04-30

**Authors:** Jessica Hack, Graham Martin

**Affiliations:** 1 Provisional Psychologist, Newcastle Mental Health Service, Newcastle, NSW 2300, Australia; jess_hack@live.com; 2 Department of Psychiatry, The University of Queensland, Royal Brisbane and Women’s Hospital, Herston, Brisbane, QLD 4066, Australia

**Keywords:** non-suicidal self-injury, adults, Shame, expressed emotion, emotion dysregulation, depression, anxiety, stress

## Abstract

A cross-sectional study examining relationships between perceived family Expressed Emotion and shame, emotional involvement, depression, anxiety, stress and non-suicidal self-injury, in 264 community and online adults (21.6% male). We compared self-injurers with non-self-injurers, and current with past self-injurers. Self-injurers experienced more family Expressed Emotion (EE) than non-injurers (*t*(254) = −3.24, *p* = 0.001), linear contrasts explaining 6% of between-groups variability (*F*(2, 254) = 7.36, *p* = 0.001, η^2^ = 0.06). Differences in EE between current and past self-injurers were not significant. Overall shame accounted for 33% of between-groups variance (*F*(2, 252) = 61.99, *p* < 0.001, η^2^ = 0.33), with linear contrasts indicating self-injurers experienced higher levels compared to non-injurers (*t*(252) = −8.23, *p* < 0.001). Current self-injurers reported higher overall shame than past self-injurers (*t*(252) = 6.78, *p* < 0.001). In further logistic regression, emotional involvement and overall shame were the only significant predictors of self-injury status. With every one-unit increase in emotional involvement, odds of currently engaging in self-injury decreased by a factor of 0.860. Conversely, a one-unit increase in overall shame was associated with an increase in the odds of being a current self-injurer by a factor of 1.05. The findings have important treatment implications for engaging key family members in intervention and prevention efforts.

## 1. Introduction

The overall lifetime prevalence of non-suicidal self-injury in Australia is 8.1% [1], with intentional self-harm accounting for about 28,000 hospital separations per annum [2]. Self-injury commonly begins in early adolescence [3], rates peak in late adolescence and young adulthood [1], but substantial numbers of adults and older Australians also self-injure [4]. Self-injury is often impulsive, can be associated with suicidality, is co-morbid with a range of psychiatric difficulties, and serves interpersonal and intrapersonal functions [5,6].

### 1.1. The Role of Family Dysfunction

Links between family dysfunction and suicidal thoughts, behaviours, and attempts have been documented within normative [7] and clinical [8] adolescent samples. Influential factors include parental separation or divorce, parental psychopathology, family history of suicide attempts, and domestic violence or abuse [9,10,11]. Impaired parent–child relationships have been implicated in suicidal behaviours [12,13,14].

In early work, the Family Assessment Device [15], suggested family dysfunction was correlated with adolescent self-harm [16]. Subsequently, the Parental Bonding Instrument [17] was used to investigate parenting style in adolescent depression and suicidality [18,19,20,21]. Associations between problematic parental bonding and non-suicidal self-injury (NSSI) are now confirmed, whereas family structure and stress appear less influential [22,23].

‘Parental Bonding’ includes level of caring, over-protectiveness, and parental criticism, and has conceptual overlap with the older construct Expressed Emotion (EE) [24], now also shown to be associated with NSSI and suicidal ideation in teenagers [25,26].

### 1.2. Expressed Emotion

The construct of EE, derived from studies on schizophrenia, aimed to clarify why some patients repeatedly relapsed after hospital discharge while others did not. Scales used to assess emotional involvement within the family found that hostility expressed towards the patient, and domineering behaviour, were likely predictors of relapse, and related to the amount of time the patient spent with key family members, as well as the overall family level of EE [24,27].

EE has been refined into two distinct components: critical comments and hostility, and emotional over-involvement [28,29]. Criticism mainly targets behaviours, whereas hostility is characterised as broader dislike or rejection of the person. Conversely, emotional over-involvement refers to exaggerated and dramatic emotional responses, and/or over-protective and intrusive behaviours. The theory posits that when an individual parent characterised by high EE believes symptoms of mental illness are within the control of their loved one, they become angered when the adolescent fails to prevent themselves from exhibiting such symptoms. This leads the parent to make repeated critical comments towards their adolescent and become emotionally over-involved in their life.

While EE is traditionally assessed via the Camberwell Family Interview [30], recent studies have turned to more cost effective, shorter measures such as the Five Minute Speech Sample (FMSS) [31], the Level of EE Scale (LEES) [32], and the Family Emotional Involvement and Criticism Scale (FEICS) [33]. To date, studies of EE and self-harming behaviours have focussed on youth populations [34], all concluding that negative family environments characterised by high levels of EE lead adolescents to feel progressively depressed, hopeless, and trapped, in turn leading them towards self-harm and suicidal ideation as a means of escape. The Camberwell Family Interview, was used with families of Portuguese youth admitted to hospital following a self-harming episode (including excessive ingestion of substances) [35]. Self-harmers came from family environments characterised by higher levels of criticism and emotional over-involvement in comparison to non-self-injuring controls.

Wedig & Nock [25] used the Five Minute Speech Sample to assess parents, with adolescents from the community and outpatient mental health clinics interviewed about self-injurious thoughts and behaviours (SITB), mental disorders, and self-criticism. High parental EE was related to the presence and frequency of suicidal ideation, attempts, plans, and NSSI. Specifically, parental criticism was associated with an increase in such behaviours, though emotional over-involvement was not. The relationship could not be accounted for by mental disorders, and findings supported a moderated model such that a stronger relationship between parental EE and SITB was displayed by adolescents with self-critical cognitive styles. These findings are supported by a study finding self-injurious behaviour in females to be related to dimensions of trait perfectionism, and parental criticism [36].

Based on these prior studies, for the present study we predicted self-injurers would come from caregiving environments characterised by higher levels of EE compared to those with no history of self-injuring. We also predicted current self-injurers would report higher levels of EE by comparison to past self-injurers.

### 1.3. Shame

Lewis describes shame as an overwhelming and debilitating emotion, negatively scrutinizing the self and seeing it as defective [37]. It is suggested shame promotes negative self-beliefs and fosters worthlessness, humiliation, and powerlessness leading people to want to ‘sink into the floor’ or disappear [38]. Individuals who experience feelings of shame believe their internal self cannot be changed. Most research focusing on shame refers to ‘Shame-Proneness’, a predisposition to experience shame across different situations [39,40]. Poor shame regulation may lead to low self-esteem or depression [37,41]. An individual perceiving the self as ‘defective’ or ‘flawed’ can become hostile and direct aggression into self-injurious behaviour to regulate shameful feelings [42].

Shame-Proneness has been found in relation to suicidal behaviours in men [43], and in female prisoners engaging in self-injurious acts on two or more occasions [44]. In female students transitioning from high school to university, self-injurers report higher levels of bodily shame, as well as shame regarding personal habits, abilities, and manners with others [36]. For the current study we predicted self-injurers would experience higher levels of shame compared to those with no history of self-injury, and also that current self-injurers would report more shame than previous self-injurers.

### 1.4. Emotion Dysregulation

The affect-regulation model of self-injury [5] suggests people engage in NSSI during emotional distress to regulate emotions in the absence of better control. Emotion regulation refers to the ability to manage and modify emotional experiences in order to make them consistent with personal objectives [45]. The construct involves awareness, understanding, and acceptance of emotions, the ability to inhibit impulsive behaviour in the face of emotional distress, the ability to engage in goal-directed behaviour, and a willingness to pursue activities that may involve the experience of negative emotions [46]. Deficits in these areas are considered indicative of emotional dysregulation—the inability to regulate emotions when experiencing periods of negative affect.

According to Linehan’s biosocial model [47], dysregulation results from a biological and environmental interaction. Vulnerability to intense emotionality mixes with an environment which poorly models ways to constructively manage emotion. In support of this, a study on male undergraduates found the largest amount of variance in self-injury frequency attributable to emotion dysregulation. Additionally, it could reliably differentiate men who self-injured frequently from those with no history of the behaviour [48].

The model was later extended to posit that ineffective emotion dysregulation fosters self-injury within social contexts that are hostile and unsupportive [49]. A recent study of adolescents demonstrated that family relational problems characterized by high conflict levels and a lack of support for managing emotions were associated with greater emotion dysregulation [50]. The current study aimed to extend this literature by investigating the effect of emotion dysregulation over levels of NSSI. We predicted that self-injurers would have higher levels of emotion dysregulation compared to their non-injuring counterparts. In addition, we hypothesised that current self-injurers would report higher levels of emotion dysregulation than previous self-injurers.

### 1.5. Depression

Depression manifests as a combination of feelings of loneliness, sadness, irritability, hopelessness, agitation, worthlessness, and guilt. If the affect-regulation model is correct, then depression may function as a precipitating factor of self-injury [5]. In a study of 53 psychiatric patients with cluster B personality disorders (i.e., Borderline Personality Disorder), those with a history of self-injury in addition to suicide attempts reported higher levels of depression, hopelessness, and aggression in comparison to those who had attempted suicide alone [51]. A non-clinical population of university undergraduates showed differences in depressive and anxious symptoms between self-injurers and a control group, but differences disappeared when symptoms of BPD were accounted for [52]. Such findings suggest that, while depression is commonly reported among self-injurers, it remains unclear if it is specific to or causal of self-injury, or just an expression of associated mental health difficulties.

Tentative support for a mediated relationship has found that family dysfunction led to higher depression levels in adolescents, which in turn contributed to self-injury, suicidal thinking, and suicide attempts [16]. Further, measures of depression may differentiate adolescents who engage in NSSI from those who self-injure with suicidal intent [53].

The current study aimed to explore this complexity in the literature by examining depression across levels of self-injury in a community sample. We predicted that individuals engaging in self-injury would report higher levels of depression than those without a self-injurious history, and individuals currently engaging in NSSI would report higher depression levels than those who had ceased self-injuring.

### 1.6. Anxiety

Self-injury can be preceded by a sense of rising tension and subsequent urges to reduce it [54]. Early literature reports subjects feeling ‘tense’ and ‘anxious’ followed by the need to ‘control their mind’ and ‘stop it from racing’, prior to engaging in self-injurious acts [55]. Higher anxiety ratings have been demonstrated in community samples of self-injuring adolescents [56]. Research on military recruits suggests anxiety maintains a unique association with self-injury over and above that of depression, and that the relationship between depression and self-injury is reduced once anxiety has been controlled for [57].

We predicted that individuals who engaged in self-injury would report higher levels of anxiety compared to those with no history of self-injury, and that current self-injurers would report higher anxiety levels in comparison to previous self-injurers.

### 1.7. Stress

It has been proposed that individuals engage in self-injury as a strategy to cease extreme and intolerable physiological arousal caused by stressful life events [58,59]. A study on clinically referred adolescents found evidence for this model by successfully predicting increased NSSI due to the interaction between negative attributional style and stressful life events, even after accounting for depressive symptoms [60]. Further, those engaging in NSSI exhibit higher levels of physiological hyperarousal when responding to stress, as well as decreased willingness to persist in tasks, and poorer distress tolerance [61].

We predicted self-injurers would experience higher levels of stress in everyday life in comparison to individuals who do not self-injure, and further that individuals currently self-injuring would report higher stress levels than those who had ceased NSSI.

Overall, the current study aimed to explore the role of EE, shame, and NSSI in a community sample of adults. Self-injury was defined as ‘the direct and deliberate injuring or destruction of body tissue in the absence of conscious suicidal intent’ [62]. This definition encompasses non-suicidal behaviours of burning, scratching, skin carving, cutting, banging, or hitting body parts; wound picking; and needle use. It is differentiated from suicide attempts and socially sanctioned forms of self-harm such as tattoos and piercings.

Differences in individual factors of emotion dysregulation, shame, depression, anxiety, and stress were investigated comparing current self-injurers, past self-injurers, and those who had never engaged in self-injury. We predicted that self-injurious individuals would come from caregiving environments characterized by high EE and would experience increased levels of emotion dysregulation, shame, and negative affect in comparison to those with no history of self-injury. Furthermore, we hypothesised current self-injurers would report higher levels of EE and all individual factors in comparison to previous self-injurers.

## 2. Materials and Methods

### 2.1. Participants and Procedures

With Ethics approval from the University of Queensland Behavioural and Social Sciences Ethical Review Committee (2012; S4200355), participants were sought from first year psychology students, expected to take part in surveys as part of their course experience. In addition, participants were sought from self-injury focussed social networking sites (on Facebook or Twitter), and online self-harm/self-injury community groups. All first year student participants met with the first researcher in person to discuss any concerns regarding the study, and were advised they could attend a debrief session to discuss the content of the study after completion. All potential subjects were provided with the ‘Qualtrics’ online survey link, instructed to read both Information and Debrief sheets, and informed they could withdraw from the study at any time without penalty or prejudice. They were assured all responses would remain confidential, and that their identity could not be linked to the survey in any way.

A total of 264 participants (57 males and 207 females, aged 14–59 years; *M =* 22.95, *SD* = 7.07) completed the questionnaire. They identified as Australian (*n* = 177, 67%), American (*n* = 18), Chinese (*n* = 14), Malaysian (*n* = 9), New Zealand (*n* = 5), English (*n* = 8), and other (*n* = 33). English was the primary language for 85.6%. Just over half the sample (52.3%) lived with their family of origin, 26.9% with friends, 15.2% with a partner, and 5.3% alone. Participants engaged in activities (e.g., outings and dinners) with their family either daily (11.4%), at least once a week (29.9%), or at least once a month (33.5%), while 21.6% did so less than once a month. Only 3.4% had no contact at all.

### 2.2. Measures

The study included demographic information, a self-injury questionnaire, and standardised scales measuring EE, shame, depression, anxiety and stress, and emotion regulation.

### 2.3. Family Emotional Involvement and Criticism Scale (FEICS) 

This 14-item self-report scale assessed levels of EE within the family environment. Participant responses ranged from 1 (almost never) to 5 (almost always) on a 5-point Likert scale, higher numbers indicating higher levels of EE [63]. Two subscales—Perceived Criticism (e.g., “My family approves of almost everything I do”) and intensity of Emotional Involvement (e.g., “I am upset if anyone else in my family is upset”)—each have seven items. Total scores for each subscale range from 7 to 49. An overall score was calculated by summing all 14 items.

Good internal consistencies for each subscale are reported with Cronbach alpha at 0.82 for PC and 0.74 for EI. The current study found slightly higher internal reliability coefficients for EI (0.75) and PC (0.85) subscales.

### 2.4. Experience of Shame Scale 

This 25-item scale assessed feelings of shame specifically related to the self, and performance abilities. Responses on a 4-point Likert scale range from 1 (not at all) to 4 (very much) at any time over the past year, higher scores indicating increased feelings of shame. There are three subscales. Characterological Shame comprises 12 items (e.g., “Have you felt ashamed of the sort of person you are?”), Behavioural Shame includes nine items (e.g., “Have you avoided contact with anyone who knew you said something stupid?”), and Bodily Shame contains four items (e.g., “Have you felt ashamed of your body or any part of it?”). Items for each area of shame address experiential, cognitive, and behavioural components. The ESS provides researchers with the opportunity to replace two ‘competition’ questions with ones about ‘failure’; this was the version used. [64]

Construct validity has been demonstrated for the total scale, and component subscales of the ESS. The current study found slightly higher internal consistency coefficients for Characterological (*a* = 0.95), Behavioural (*a* = 0.93), and Bodily (*a* = 0.89) Shame, compared to the original study Cronbach alphas of 0.90, 0.87, and 0.86 respectively [64].

### 2.5. Depression Anxiety Stress Scale 21 (DASS-21) 

This scale has 21 self-report items with three subscales containing seven statements each: Depression (e.g., “I couldn’t seem to experience any positive feeling at all”), Anxiety (e.g., “I felt scared without any good reason”), and Stress (e.g., “I found it hard to wind down”). Responses are assessed on 4-point Likert scales relating to the previous 30 days. Scores range from 0 (did not apply to me at all) to 3 (applied to me very much or most of the time). Higher scores indicate higher depression, anxiety, or stress. Internal consistencies (coefficient alpha) for each scale for the DASS normative sample were Depression 0.91, Anxiety 0.84, and Stress 0.90. [65]. The current study found comparable alphas of 0.94, 0.89, and 0.91, respectively.

### 2.6. Difficulties in Emotion Regulation Scale (DERS) 

Participants’ rated how often each item applied to them using a 5-point Likert scale (1 (almost never) to 5 (almost always)), higher scores suggesting greater emotion dysregulation. There are six subscales. Non-acceptance of emotional responses (e.g., “When I’m upset, I become angry at myself for feeling that way”), Inability to engage in goal-directed behaviour when distressed (e.g., “When I’m upset, I have difficulty focusing on other things”), Impulse control difficulties when distressed (e.g., “When I’m upset, I feel out of control”), Limited access to strategies for effective regulation (e.g., “When I’m upset, I start to feel very bad about myself”), Lack of emotional awareness (e.g., “I am attentive to my feelings”), and Lack of emotional clarity (e.g., “I am confused about how I feel”). Of the 36 items, 11 are reverse-scored [46].

The DERS has good test–retest reliability (0.88), and high internal consistency (Cronbach alpha 0.93 for the overall scale, with subscale reliabilities each exceeding 0.80) [46]. The current study overall internal consistency was 0.91, though subscales ranged from 0.63 to 0.93.

### 2.7. Deliberate Self-Injury Questionnaire 

This measure provides a working definition of deliberate self-injury, followed by questions about frequency, methods, and purpose of self-harming behaviours. Additional items ask about suicidality, receiving medical treatment or supportive counselling for injuries, as well other factors that encourage cessation [66].

Participants were classified into one of three groups; ‘no history of deliberate self-injury’ (control group), ‘currently self-injuring’, or ‘previous history of deliberate self-injury’. The first question is “Have you ever engaged in deliberate self-injury? If only once, please still select yes”. Those answering ‘No’ were allocated to the control group and diverted automatically to the last standardised scale. Those answering ‘Yes’ completed the remainder of the survey. The second question, “Do you currently (at least once in the past 12 months) engage in self-injury?” separated the remaining participants into those responding ‘Yes’ (the ‘currently self-injuring’ group), and those answering ‘No’ (the ‘previous history of deliberate self-injury’ group).

## 3. Results

All statistical analyses were completed in SPSS 20.0 (IBM SPSS Statistics for Windows, Version 20.0. Armonk, NY: IBM Corp, USA) provided by the University of Queensland. Frequency distributions revealed perceived criticism, characterological shame, overall psychological distress, depression, anxiety, and emotion dysregulation were significantly skewed (*z* > ±3.29). All analyses containing these variables used square root transformations to eliminate skewness. Overall, EE had a medium correlation with perceived criticism (*r* = 0.67; *p* < 0.001) and a small to medium correlation with emotional involvement (*r* = 0.49; *p* < 0.001).

### 3.1. Non-Suicidal Self-Injury 

A lifetime history of NSSI was reported by 121 participants, 109 females (41.3%) and 12 males (4.5%). Of these, 7.6% self-injured daily, 17.8% weekly or monthly, 3.8% every 3 months, 7.2% at least twice a year, and 9.1% had only engaged in one self-injurious act. Participants were grouped into three categories: Current Self-Injury (*n* = 67), Past Self-Injury (*n* = 54), and No History of Self-Injurious Behaviour (*n* = 143). Attempted suicides were reported by 19.8% (*n* = 24); all from the current and previous self-injury groups. Females were more likely to belong to one of the self-injury groups (χ^2^ (2, *N* = 264) = 18.06, *p* < 0.001, Φ = 0.26). Groups did not differ by age (χ^2^ (62, *N* = 262) = 68.21, *p* = 0.27, Φ = 0.36), country of birth (χ^2^ (84, *N* = 264) = 105.40, *p* = 0.06, Φ = 0.44), or language spoken (χ^2^ (42, *N* = 264) = 47.89, *p* = 0.25, Φ = 0.30).

Most self-injuring participants reported ‘cutting’ (89, 73.6%), with 45 (35.5%) ‘punching or hitting of self’, 31 (25.6%) ‘skin carving’, and 27 (22.3%) ‘purging’. Other self-injury reported included ‘scratching’, ‘hair pulling’, ‘starvation’, and ‘picking at skin’. Medical treatment for self-injury was sought by just over half, with 37 self-injurers consulting a general practitioner (30.6%) and a further 26 attending their local emergency department (21.5%).

### 3.2. Functions of Self-Injury

Several affect regulation functions for NSSI were endorsed, including ‘to alleviate and control aversive affective arousal’, ‘to release all the emotion built up inside’, ‘to alleviate feelings of sadness or anxiety’, ‘to make the inside match the outside’, and ‘the release of tension/stress’. Other reasons included ‘to punish myself for a failure’, ‘I enjoy the sensation’, and ‘to make my heart race’ (sensation seeking); ‘for attention’ and ‘to tell people I was sad in hope that people would take care of me when they saw the wounds’ (interpersonal influence).

### 3.3. Self-Injury Cessation

Of participants with a lifetime history of NSSI, 87% reported that over a year had passed since last engaging in self-injury. Influences identified by participants included supportive therapy or counselling (*n* = 13), support from family and friends (*n* = 24), support from a significant other (*n* = 10), and finding a meaning in life (*n* = 21). Additional reasons included ‘getting older and finding new ways to cope with things’, ‘found new confidence’, and ‘anxiety of people finding out’.

### 3.4. Group Comparisons

To investigate significant between group differences, a series of one-way ANOVAs were conducted. Preliminary data checks revealed that the current sample violated the ANOVA assumption of homogeneity of variances. Welch’s F is reported where appropriate. Where necessary, contrast tests are reported with equal variance not assumed. To reduce Type 1 and ‘familywise’ error, a conservative *p <* 0.01 significance level was adopted for all tests. Effect sizes were calculated using Cohen’s *d*. As seen in Table 1, significant differences were found for all dependent measures between the three focal groups.

Given the violation of the ANOVA assumption of homogeneity of variances significant findings were followed up using linear contrasts. Please see Table 2.

### 3.5. Expressed Emotion

Overall EE differed significantly between the three focal groups, explaining 6% of between-groups variability (*F*(2, 254) = 7.36, *p* = 0.001, η^2^ = 0.06). Linear contrasts revealed that self-injuring individuals reported significantly more overall EE than non-injuring participants (*t*(254) = −3.24, *p* = 0.001). Differences between current and past self-injurers on overall EE did not reach significance at *p* < 0.01, (*t*(254) = 1.78, *p* = 0.076).

### 3.6. Perceived Criticism.

The three focal groups differed significantly, with perceived criticism accounting for 17% of between-groups variance (*F*(2, 111.49) = 15.48, *p* < 0.001, η^2^ = 0.17). Linear contrasts indicated individuals engaging in NSSI reported significantly more perceived criticism from family members than individuals with no history of NSSI (*t*(226.37) = −3.68, *p* < 0.001). Current self-injurers reported significantly more perceived criticism from family members compared to those who had ceased such behaviours (*t*(117.52) = 4.31, *p* < 0.001).

### 3.7. Emotional Involvement

The significant differences in emotional involvement between the three groups explained 9% of the total between-groups variability (*F*(2, 258) = 13.42, *p* < 0.001, η^2^ = 0.09). Linear contrasts revealed individuals with no history of self-injury reported significantly more emotional involvement from family members than self-injurers overall (*t*(258) = 2.96, *p* = 0.003). Past self-injurers reported significantly more emotional involvement from family members than current self-injurers (*t*(258) = −3.98, *p* < 0.001).

Levels of overall shame accounted for 33% of between-groups variance (*F*(2, 252) = 61.99, *p* < 0.001, η^2^ = 0.33). Linear contrasts indicated that self-injuring individuals reported significantly higher levels of overall shame compared to non-injurers (*t*(252) = −8.23, *p* < 0.001). Current self-injurers reported significantly higher levels of overall shame than did previous injurers (*t*(252) = 6.78, *p* < 0.001).

The three focal groups differed significantly in levels of Characterological Shame explaining 24% of between-group variability (*F*(2, 258) = 54.61, *p* < 0.001, η^2^ = 0.24). Linear contrasts revealed that individuals engaging in NSSI reported significantly higher levels than non-self-injurers (*t*(258) = −7.85, *p* < 0.001), and current self-injurers reported significantly higher levels of Characterological Shame than past self-injurers (*t*(258) = 6.30, *p* < 0.001).

Similarly, significant differences in Behavioural Shame were found between the three groups accounting for 25% of the total between-groups variance (*F*(2, 256) = 43.00, *p* < 0.001, η^2^ = 0.25), with linear contrasts indicating self-injurers reported significantly more than those without a history of self-injury (*t*(256) = −6.92, *p* < 0.001). Those currently engaging in NSSI reported significantly higher levels than past self-injurers (*t*(256) = 5.58, *p* < 0.001).

Levels of Bodily Shame showed a similar pattern, differing significantly across levels of self-injury explaining 24% of the between-groups variability (*F*(2, 129.95) = 49.08, *p* < 0.001, η^2^ = 0.24). Individuals engaging in NSSI reported significantly more Bodily Shame than those without a history of NSSI engagement (*t*(241.45) = −7.92, *p* < 0.001), with current self-injurers reporting significantly more than past self-injurers (*t*(100.52) = 4.31, *p* < 0.001).

### 3.8. Psychological Distress

Overall, psychological distress differed significantly across the three focal groups accounting for 26% of total between-groups variance (*F*(2, 107.33) = 68.30, *p* < 0.001, η^2^ = 0.26). Linear contrasts revealed that self-injurers reported significantly higher levels of psychological distress compared to non-injurers (*t*(215.12) = −9.37, *p* < 0.001), with current self-injurers reporting significantly higher levels of psychological distress than past self-injurers (*t*(109.15) = 6.37, *p* < 0.001).

### 3.9. Depression

Significant differences in depression were found across levels of self-injury explaining 27% of between-group variability (*F*(2, 109.49) = 56.92, *p* < 0.001, η^2^ = 0.27). Linear contrasts indicated that self-injurers reported significantly more depression than non-injurers (*t*(220.93) = −8.22, *p* < 0.001), and current self-injurers reported significantly more depression than past self-injurers (*t*(113.36) = 6.53, *p* < 0.001).

### 3.10. Anxiety

Anxiety differed significantly across levels of self-injury, accounting for 18% of total between groups variance (*F*(2, 109.88) = 48.72, *p* < 0.001, η^2^ = 0.18); self-injurers significantly more anxious than non-injurers (*t*(221.48) = −8.43, *p* < 0.001), and current self-injurers significantly more anxious than previous self-injurers (*t*(116.75) = 5.12, *p* < 0.001).

### 3.11. Stress

Levels of stress accounted for 34% of between-groups variance (*F*(2, 258) = 65.45, *p* < 0.001, η^2^ = 0.34), with linear contrasts showing self-injurers reporting significantly more than non-self-injurers (*t*(258) = −9.16, *p* < 0.001), and current self-injurers reporting significantly more than previous self-injurers (*t*(258) = 6.09, *p* < 0.001).

#### Emotion Dysregulation

Emotion dysregulation differed significantly across the groups accounting for 11% of between groups variability (*F*(2,245) = 55.02, *p* < 0.001, η^2^ = 0.11). Self-injurers reported significantly higher levels than those without a history of such behaviours (*t*(245) = −7.89, *p* < 0.001), and current self-injurers reported higher levels compared to previous self-injurers (*t*(245) = 6.33, *p* < 0.001).

### 3.12. Logistic Regression

Given the repeated pattern of significant difference between the three groups of interest on each of our variables, two binary logistic regressions analyses were completed to predict the relative importance of each predictor variable, and to further assess self-injury group membership. The first model focused on self-injuring individuals compared to those with no history of self-injury and contained all five predictors: perceived criticism, emotional involvement, shame, psychological distress, and emotion dysregulation. A total of 225 cases were analysed and the full model was statistically significant (χ^2^ (5, *N* = 225) = 73.59, *p* < 0.001). The model accounted for between 27.9% and 37.3% of variance in self-injury behaviour, with 60.4% of self-injurers successfully predicted, and 83.9% of predictions accurate for non-self-injurers. Overall, 73.3% of predictions were correctly classified. Table 3 shows coefficients and Wald statistics, as well as associated probability values and degrees of freedom for each of the predictor variables. In logistic regression, overall psychological distress was the only significant predictor of self-injury group membership. The coefficients indicate that for every one-unit increase in overall psychological distress, the odds of engaging in self-injury increases by a factor of 1.50.

A second logistic regression was conducted to determine if any single predictor could distinguish between current and previous self-injurers. A total 101 cases were analysed and the full model containing all five predictors was statistically significant (χ^2^ (5, *N* = 101) = 45.87, *p* < 0.001), accounting for between 36.5 and 48.8% of the variance in self-injury behaviour, with 80.0% of current self-injurers successfully predicted, and 73.9% of predictions accurate for previous self-injurers. Overall, 77.2% of predictions were correctly classified. Table 4 displays the coefficients and Wald statistics, as well as associated probability values and degrees of freedom for each predictor variable. Emotional involvement and overall shame were the only significant predictors of self-injury status. The coefficients indicate that for every one-unit increase in emotional involvement, the odds of currently engaging in self-injury decrease by a factor of 0.860, and that a one-unit increase in overall shame is associated with an increase in the odds of being a current self-injurer by a factor of 1.05.

Overall EE had a medium correlation with perceived criticism (*r* = 0.67; *p* < 0.001) and a small to medium correlation with emotional involvement (*r* = 0.49; *p* < 0.001). A small negative correlation was found between EE and perceived criticism (*r* = −0.31; *p <* 0.001). These relationships are inconsistent with previous work [25] which found non-significant correlations between EE and EI, and EI and PC.

## 4. Discussion

Despite the cross-sectional nature of this research with adults, we sought to specifically examine the effect of EE on NSSI behaviour, and whether shame contributed to the risk of continuing NSSI. Group differences were investigated between current, past, and non-self-injurers. Overall, self-injurers reported higher levels of family EE compared to those with no history of NSSI, and current self-injurers reported significantly higher levels than those who had successfully given up self-injury. Overall, psychological distress, shame, and emotional involvement were unique predictors of self-injury group membership. The strengths and limitations of the research are discussed and implications for clinical practice and recommendations for future research are provided.

### 4.1. Non-Suicidal Self-Injury

With 45.8% of participants engaging in NSSI, prevalence rates were similar to some prior research [67], but higher than the majority of previous studies. This is due to our recruitment techniques, with part of the sample accessed through social networking sites and online self-harm/self-injury community groups, the high percentage of self-injurers in our sample enabling us to highlight differences between current and previous self-injurers.

Self-injurious behaviour was reported by more females than males, and while this supports other studies, for example, [68,69], we must stress that our study is not an epidemiological study and therefore comparative gender rates are meaningless compared with large scale epidemiological surveys which have found comparable prevalence rates among males and females [1].

### 4.2. Functions

The current study found support for four of the seven previously identified functions of self-injury. The most commonly reported reasons for self-injury supported the affect-regulation and self-punishment models [47], as well as anti-dissociation and sensation-seeking [5]. No support was found for the anti-suicidal model [5] nor the interpersonal-boundaries model [70].

#### Expressed Emotion, Perceived Criticism and Emotional Involvement

Previous research has found high levels of EE within the family environment to be associated with self-harm and suicidal ideation in youth populations [34]. In our non-clinical adult sample, in line with predictions, self-injurers also reported higher levels of overall EE and greater perceived criticism (PC) compared to those with no history of self-injury, supporting previous research [25,35]. Current self-injurers reported more perceived criticism from family members compared to those who had ceased, even though logistic regression suggested that neither EE nor PC levels discriminate between current and previous self-injury groups.

Of note, individuals who had never engaged in NSSI reported more emotional involvement (EI) from family members by comparison to self-injurers, and this needs to be explained. The Family Emotional Involvement and Criticism Scale [63] does not prejudge emotional involvement by assuming that high intensity levels are indicative of detrimental outcomes, but measures intensity of emotional involvement perceived by participants, as opposed to the level of emotional over-involvement displayed by family members. There were no differences in the lower levels of overall emotional involvement (EI) found between current and previous self-injurers.

It is possible that the current study contradicts prior research on EE and self-injury because it utilised a broad adult sample rather than youth populations. As just under half the sample lived away from home, and around a third reported only spending time with their family on a monthly basis, the frequency of relative contact may have moderated this effect.

### 4.3. Individual Risk Factors

**Emotion dysregulation.** The theory suggests that emotion dysregulation fosters self-injury in hostile and unsupportive social contexts as a result of an interaction between biological vulnerability and poorly modelled emotion management skills [47,49]. The current study aimed to extend research in this area by investigating the effect of emotion dysregulation across levels of self-injury in an adult community sample. As predicted, findings revealed greater emotion dysregulation for self-injurers than for individuals with no history of NSSI engagement. Current self-injurers reported greater emotion dysregulation compared to those who had a past history of self-injuring. Despite the gender differences between studies, these findings support other research findings that emotion dysregulation accounted for a large proportion of self-injury variance in undergraduate males [48].

Higher levels of emotion dysregulation found in self-injurers supports the idea that NSSI is a maladaptive coping mechanism used by individuals in an attempt to regulate periods of negative affect. Other work has demonstrated that adolescents with higher levels of emotion dysregulation were from family environments characterised by high conflict [50]. This was reflected in our participant’s free responses when describing the purpose of self-injury (e.g., “to alleviate anger or times of overwhelming emotion that I don’t have the skills to cope with” or “I would usually do it when having a fight with someone and feeling helpless in the situation”). It is plausible that emotion dysregulation may precede self-injury or mediate the association between family EE and NSSI. Longitudinal research is required to investigate these relationships further.

**Shame**. The current study aimed to build on existing literature by investigating the association between shame and NSSI in a community sample of adults. Previous research indicates that individuals who experience overwhelming levels of shame are prone to viewing themselves as defective and worthless, become self-loathing, and cope by directing their aggression towards their external self [37,42]. Recent research suggests those reporting higher levels of bodily shame, and more shame related to their scars may be at higher risk of repetition of self-injury [71,72].

Free text reasons our participants gave for self-injuring included “self-sabotage/hatred” and “punishment for crippling self-loathing”. These feelings may result from shame of ones’ body, character or behaviour, and have been linked to a variety of negative psychological outcomes [38].

We examined overall shame, characterological shame, behavioural shame and bodily shame. As predicted, self-injurers reported higher levels of shame across all four categories by comparison to non-self-injurers. Current self-injurers reported higher levels of shame across all categories compared to past self-injurers. These results support work [36] in which self-injuring female students transitioning from school to university reported higher levels of shame over their bodies, mannerisms and personal habits. Results from our logistic regression suggest that overall shame has unique predictive power to differentiate current from previous self-injurers. Taken together, these findings suggest that reducing levels of shame for an individual may aid in cessation of self-injury. We recommend future research investigate the differential effects of overall, bodily, behavioural and characterological shame in order to develop viable treatments that target these issues in self-injurers.

**Depression.** We assessed the influence of depression in a non-suicidal community sample of adult self-injurers. In line with predictions, self-injurers reported higher levels of depression compared to those with no self-injury history. In addition, follow-up tests revealed that current self-injurers reported higher levels of depression compared to those who had given up self-injury. In support of this, participants’ free-response text indicated that feelings of depression precipitated self-injury (e.g., “Depression coupled with a recent breakup” and “a feeling of hopelessness, as though my life has no purpose”). These results conflict with studies suggesting that while self-injurers commonly report depression, they do not differ on severity compared to non-injuring controls [73,74]. Even though depression was not a unique predictor of self-injury in our logistic regression, it clearly remains an important target in therapeutic approaches with self-injurers.

**Anxiety**. Our study built on existing research suggesting self-injurious acts are a maladaptive way of coping with feelings of rising tension and anxiousness [55]. This was confirmed in free text responses reporting that self-injury was “to release feelings of anxiety” and because “every now and then when I got anxious I would find myself doing it”.

As predicted, the DASS scores for anxiety in self-injurers were significantly higher than for individuals with no history of NSSI. Further, current self-injurers reported higher anxiety levels by comparison to previous self-injurers. These results in adults support a study that found higher anxiety levels in an adolescent community sample of self-injurers [56].

Even though anxiety was not a unique predictor of self-injury in logistic regression, responses obtained from the current study suggest that equipping anxious individuals with the skills to decrease and regulate their anxiety appropriately may lead to cessation of self-injury or prevent it from occurring in those at risk.

**Stress**. Research in self-injuring populations suggests individuals self-injure after experiencing stressful and adverse life events that cause intolerable physiological arousal [59]. Our findings from the DASS Stress scale confirm self-injurers experience higher levels of stress compared to their non-injuring counterparts. In addition, current self-injurers reported higher stress levels compared to those who had given up self-injury. The relationship was further supported by participants’ free-text responses in which they attributed the purpose of their self-injury to dealing with “Stress with schoolwork overload” and to “release tension/stress”. These findings support findings that adolescents with greater physiological hyperarousal when responding to stress were more likely to engage in NSSI [61].

Psychological Distress. The Depression Anxiety Stress Scale (DASS) [65] allows for an overarching factor of psychological distress to be calculated. As perhaps expected given scores on individual constructs of depression, anxiety and stress, self-injurers had greater levels of overall psychological distress, and current self-injurers had greater levels of psychological distress compared to past self-injurers. In logistic regression, psychological distress was able to uniquely distinguish self-injurers from those who had never engaged in NSSI.

To our knowledge, this is the first study to directly assess the impact of EE, and resulting shame, in NSSI in a broad community sample of adults. While the level of psychological distress overall (including depression, anxiety and stress) was able to distinguish self-injurers from non-self-injurers in logistic regression, overall shame showed unique predictive power in differentiating current from previous self-injurers.

This study was also the first to assess the relationship between EE and NSSI by using a self-report measure to investigate the level of criticism and emotional involvement perceived by participants, as opposed to studies using observers to objectively rate participants’ family members. Using this measure in an online survey permitted participants to answer the questionnaire anonymously and in their own time. Participants may have been able to answer sensitive questions about the shame of their self-injurious behaviour, where they may not have felt comfortable detailing their history to a researcher.

The current study is not without limitations to be considered when interpreting and comparing results. First, the data was obtained through a cross-sectional design. Causal inferences are difficult to draw, and directionality of results may not be clear. Longitudinal studies are needed to ascertain whether a reduction in EE, shame, emotion dysregulation, depression, anxiety, and stress can permanently impact the cessation of self-injury.

Further, overall EE accounted for only 6% of variance in self-injury behaviour, meaning small differences were found between the three focal groups. It is possible that overall EE may have been underrepresented due to problems with measurement reliability. In addition, the sample consisted of adults who may have inferred ‘family’ as current partners and children instead of family of origin. As the referent environment was not specified, results must be interpreted with caution.

Finally, some participants were sourced from social media and online self-injury forums that could have biased the sample towards more serious self-injurers. Future research could use a broader recruitment process to focus on the impact of EE across all levels of self-injury within the general population.

#### Implications and Future Research

The current study provides further evidence that negative family environments characterised by high levels of overall EE and criticism play a role in the development and continuation of NSSI. Our results indicate that interactions within the familial environment can develop or worsen shame, but also the results provide support for the protective role of family members. Our findings suggest the family environment should be assessed when dealing with self-injurers.

Involving family members in treatment allows for psychoeducation alongside improvement in communication and the development of problem-solving skills. As emotional involvement had the unique ability to distinguish between current and previous self-injurers, therapy might focus on increasing the intensity of emotional involvement from family members while reducing criticism.

If family members do not wish to participate in therapy sessions, it is vital self-injurers are taught necessary skills to deal with high EE environments, as well as psychological distress and shame. Future research could focus on controlled trials of the additive role of family involvement in achieving more successful therapy outcomes.

## 5. Conclusions

This study was the first to investigate the effect of EE within caregiving environments and its impact on NSSI in a broad non-clinical adult sample. As far as possible in a cross-sectional study, we aimed to clarify the role of emotion dysregulation, shame, depression, anxiety, and stress on the developmental trajectory of self-injury. In line with predictions, self-injurers reported greater levels of overall family EE and perceived criticism compared to those with no history of NSSI. Current self-injurers reported higher levels of perceived criticism than past self-injurers, but no differences were found in overall EE. Past self-injurers reported more intense emotional involvement from family members than did current self-injurers. Levels of psychological distress, overall shame and emotional involvement were able to uniquely distinguish current from past self-injurers.

The current study has addressed gaps in the self-injury literature regarding the role of family. Our results may inform prevention and treatment strategies, and it is hoped that these findings will be incorporated into future research investigating interventions for NSSI.

## Figures and Tables

**Table 1:** Group comparisons and effect sizes (Cohen’s d).

Variable	Construct	Self-Injury Group Means					Effect Size
		Current	Previous	None	*F*	*p*	*D*
Expressed Emotion	Overall	39.58	37.53	36.01	7.36	0.001	0.06
	Perceived Criticism	20.06	14.67	13.02	15.48	0.000	0.17
	Emotional Involvement	19.52	22.98	22.99	13.42	0.000	0.09
Shame	Overall	80.73	61.08	54.84	61.99	0.000	0.33
	Characterological	36.72	26.89	23.82	54.61	0.000	0.24
	Behavioural	30.35	23.81	21.62	43.00	0.000	0.25
	Bodily	13.69	11.40	9.39	49.08	0.000	0.24
Psychological Distress	Overall	117.14	83.50	70.38	68.30	0.000	0.26
	Depression	40.86	26.91	23.19	56.92	0.000	0.27
	Anxiety	34.67	25.19	20.80	48.72	0.000	0.18
	Stress	41.76	31.28	25.86	64.45	0.000	0.34
Emotion Dysregulation		116.50	101.75	91.24	55.02	0.000	0.11

**Table 2:** Follow up linear contrasts for significant findings.

Variable	Comparison					
	Current SI & Previous SI vs. No History			Current SI vs. Previous SI		
	*t*	*p*	Effect Size	*t*	*p*	Effect Size
Overall Expressed Emotion	−3.24	0.001	0.20	1.78	0.076	-
Perceived Criticism	−3.69	0.000	0.24	4.31	0.000	0.37
Emotional Involvement	2.96	0.003	0.18	−3.98	0.000	0.24
Overall Shame	−8.23	0.000	0.46	6.78	0.000	0.39
Characterological Shame	−7.85	0.000	0.44	6.30	0.000	0.37
Behavioural Shame	−6.92	0.000	0.40	5.58	0.000	0.33
Bodily Shame	−7.92	0.000	0.45	4.31	0.000	0.39
Psychological Distress	−9.37	0.000	0.54	6.37	0.000	0.52
Depression	−8.22	0.000	0.48	6.53	0.000	0.52
Anxiety	−8.43	0.000	0.49	5.12	0.000	0.43
Stress	−9.16	0.000	0.50	6.09	0.000	0.35
Emotion Dysregulation	−7.89	0.000	0.45	6.33	0.000	0.37

NB. ‘SI’ = Self-injury.

**Table 3:** Logistic regression predicting likelihood of self-injury engagement.

Variable	*B*	SE	Wald	*df*	*p*	Exp(*β*)	95% CI for Exp(*β*)	
							Lower	Upper
Perceived Criticism	0.38	0.28	1.84	1	0.175	1.47	0.85	2.55
Emotional Involvement	−0.06	0.04	3.12	1	0.077	0.94	0.88	1.01
Overall Shame	0.02	0.01	1.64	1	0.200	1.02	0.99	1.05
Psychological Distress	0.40	0.17	5.84	1	0.016 *	1.50	1.08	2.08
Emotion Dysregulation	0.24	0.23	1.14	1	0.286	1.27	0.82	1.98
Constant	−7.45	1.91	15.34	1	0.000	0.001		

Note. * *p <* 0.05.

**Table 4:** Logistic regression differentiating current from previous self-injurers.

Variable	*B*	SE	Wald	*df*	*p*	Exp(*β*)	95% CI for Exp(*β*)	
							Lower	Upper
Perceived Criticism	0.30	0.43	.49	1	0.484	1.35	0.58	3.12
Emotional Involvement	−0.15	0.06	6.10	1	0.014 *	0.86	0.76	0.97
Overall Shame	0.05	0.02	4.95	1	0.026 *	1.05	1.01	1.10
Psychological Distress	0.49	0.26	3.75	1	0.053	1.64	0.99	2.70
Emotion Dysregulation	−0.47	0.36	1.75	1	0.186	0.62	0.31	1.26
Constant	−1.38	3.03	0.21	1	0.649	0.25		

Note. * *p* < 0.05.

## References

[1] Martin G., Swannell S., Hazell P., Harrison J., Taylor A. (2010). Self-injury In Australia: A community survey. Med. J. Aust..

[2] Australian Institute of Health and Welfare (2012). Admitted Patient Care: Overview. http://www.aihw.gov.au/haag09-10/admitted-patient-care-overview/.

[3] Andrews T., Martin G., Hasking P., Page A. (2014). Predictors of onset for non-suicidal self-injury within a School-Based sample of adolescents. Prev. Sci..

[4] Martin G., Swannell S. (2016). Non-Suicidal Self-Injury in the over 40s: Results from a Large National Epidemiological Survey. Epidemiology.

[5] Klonsky E.D. (2007). The functions of deliberate self-injury: A review of the evidence. Clin. Psychol. Rev..

[6] Muehlenkamp J.J., Claes L., Havertape L., Plener P.L. (2012). International prevalence of adolescent non-suicidal self-injury and deliberate self-harm. Child Adolesc. Psychiatry Ment. Health.

[7] Rubenstein J.L., Heeren T., Housman D., Rubin C., Stechier G. (1989). Suicidal behavior in “normal” adolescents: Risk and protective factors. Am. J. Orthopsychiatry.

[8] Miller M.L., Chiles J.A., Barnes V.E. (1982). Suicide attempters within a delinquent population. J. Consult. Clin. Psychol..

[9] Brent D.A., Perper J.A., Moritz G., Allman C., Friend A., Roth C., Baugher M. (1994). Familial risk factors for adolescent suicide: A case–control study. Acta Psychiatr. Scand..

[10] Gould M.S., Fisher P., Parades M., Flory M., Shaffer D. (1996). Psychosocial risk factors for child and adolescent completed suicide. Arch. Gen. Psychiatry.

[11] Grossman D.C., Milligan C., Deyo R.A. (1991). Risk factors for suicide attempts among Navajo adolescents. Am. J. Public Health.

[12] Adams D.M., Overholser J.C., Lehnert K.L. (1994). Perceived family functioning and adolescent suicidal behaviour. J. Am. Acad. Child Adolesc. Psychiatry.

[13] Martin G. (1996). Reported family dynamics, sexual abuse and suicidal behaviors in community adolescents. Arch. Suicide Res..

[14] Wagner B.M. (1997). Family risk factors for child and adolescent suicidal behaviour. Psychol. Bull..

[15] Byles J., Byrne C., Boyle M.H., Offord D. (1988). Ontario Child Health Study: Reliability and Validity of the General Functioning Subscale of the McMaster Family Assessment Device. Fam. Process.

[16] Martin G., Rozanes P., Allison S., Pearce C. (1995). Adolescent suicide, depression and family dysfunction. Acta Psychiatr. Scand..

[17] Parker G., Tupling H., Brown L.B. (1979). A Parental Bonding Instrument. Br. J. Med. Psychol..

[18] Martin G., Waite S. (1994). Parental bonding and vulnerability to adolescent suicide. Acta Psychiatr. Scand..

[19] Allison S., Pearce C., Martin G., Miller K., Long R. (1995). Parental influence, pessimism and adolescent suicidality. Arch. Suicide Res..

[20] Martin G., Bergen H., Allison S., Roeger L. (2004). Depression in young adolescents: Investigations using 2 and 3 factor versions of the Parental Bonding Instrument (PBI). J. Nerv. Ment. Dis..

[21] Rigby K., Slee P., Martin G. (2007). Implications of inadequate parental bonding and peer victimization for adolescent mental health. J. Adolesc..

[22] Baetens I., Claes L., Martin G., Onghena P., Grietens H., Van Leeuwen K., Pieters C., Wiersema J., Griffith J. (2014). Is non-suicidal self-injury associated with parenting and family factors?. J. Early Adolesc..

[23] Baetens I., Andrews V., Claes L., Martin G. (2015). The association between family functioning and NSSI in adolescence: The mediating role of depressive symptoms. Fam. Sci..

[24] Brown G.W., Monck E.M., Carstairs G.M., Wing J.K. (1962). Influence of family life on the course of schizophrenic illness. Br. J. Prev. Soc. Med..

[25] Wedig M.M., Nock M.K. (2007). Parental expressed emotion and adolescent self-injury. J. Am. Acad. Child Adolesc. Psychiatry.

[26] Baetens I., Claes L., Hasking P., Smits D., Grietens H., Onghena P., Martin G. (2015). The Relationship between Parental Expressed Emotions and Non-suicidal Self-injury: The Mediating Roles of Self-criticism and Depression. J. Child Fam. Stud..

[27] Brown G.W., Birley J.L.T., Wing J.K. (1972). Influence of family life on the course of schizophrenic disorders: A replication. Br. J. Psychiatry.

[28] Hooley J.M. (1985). Expressed emotion: A review of the critical literature. Clin. Psychol. Rev..

[29] Hooley J.M. (2007). Expressed emotion and relapse in psychopathology. Annu. Rev. Clin. Psychol..

[30] Vaughn C., Leff J. (1976). The measurement of expressed emotion in the families of psychiatric patients. Br. J. Soc. Clin. Psychol..

[31] Magana A.B., Goldstein M.J., Karno M., Miklowitz D.J., Jenkins J., Falloon I.R.H. (1986). A brief method for assessing expressed emotion in relatives of psychiatric patients. Psychiatry Res..

[32] Cole J.D., Kazarian S.S. (1988). The Level of Expressed Emotion Scale: A new measure of expressed emotion. J. Clin. Psychol..

[33] Shields C.G., Franks P., Harp J.J., McDaniel S.H., Campbell T.L. (1992). Development of the Family Emotional Involvement and Criticism Scale (FEICS): A self-report scale to measure Expressed Emotion. J. Marital Fam. Ther..

[34] Michelson D., Bhugra D. (2012). Family environment, expressed emotion and adolescent self-harm: A review of conceptual, empirical, cross-cultural and clinical perspectives. Int. Rev. Psychiatry.

[35] Santos J.C., Saraiva C.B., De Sousa L. (2009). The role of expressed emotion, self-concept, coping, and depression in parasuicidal behavior: A follow-up study. Arch. Suicide Res..

[36] Flett G.L., Goldstein A.L., Hewitt P.L., Wekerle C. (2012). Predictors of deliberate self-harm behavior among emerging adolescents: An initial test of a self-punitiveness model. Curr. Psychol..

[37] Lewis H.B. (1971). Shame and Guilt in Neurosis.

[38] Tangney J.P., Dearing R.L. (2002). Shame and Guilt.

[39] Tangney J.P., Wagner P.E., Gramzow R. (1992). Proneness to shame, proneness to guilt, and psychopathology. J. Abnorm. Psychol..

[40] Tangney J.P. (1996). Conceptual and methodological issues in the assessment of shame and guilt. Behav. Res. Ther..

[41] Schoenleber M., Berenbaum H. (2012). Shame regulation in personality pathology. J. Abnorm. Psychol..

[42] Brown M.Z., Linehan M.M., Comtois K.A., Murray A., Chapman A.L. (2009). Shame as a prospective predictor of self-inflicted injury in borderline personality disorder: A multi-modal analysis. Behav. Res. Ther..

[43] Lester D. (1998). The association of shame and guilt with suicidality. J. Soc. Psychol..

[44] Milligan R.J., Andrews B. (2005). Suicidal and other self-harming behaviour in offender women: The role of shame, anger and childhood abuse. Leg. Criminol. Psychol..

[45] Thompson R.A. (1994). Emotion regulation: A theme in search of definition. Monogr. Soc. Res. Child Dev..

[46] Gratz K.L., Roemer L. (2004). Multidimensional assessment of emotion regulation and dysregulation: Development, factor structure, and initial validation of the difficulties in emotion regulation scale. J. Psychopathol. Behav. Assess..

[47] Linehan M.M. (1993). Cognitive-Behavioral Treatment for Borderline Personality Disorder.

[48] Gratz K.L., Chapman A.L. (2007). The role of emotional responding and childhood maltreatment in the development and maintenance of deliberate self-harm among male undergraduates. Psychol. Men Masc..

[49] Crowell S.E., Beauchaine T.P., Linehan M.M. (2009). A biosocial developmental model of borderline personality: Elaborating and extending Linehan’s theory. Psychol. Bull..

[50] Adrian M., Zeman J., Erdley C., Lisa L., Sim L. (2010). Emotional dysregulation and interpersonal difficulties as risk factors for nonsuicidal self-injury in adolescent girls. J. Abnorm. Child Psychol..

[51] Stanley B., Gameroff M.J., Michalsen V., Mann J.J. (2001). Are suicide attempters who self-mutilate a unique population?. Am. J. Psychiatry.

[52] Andover M.S., Pepper C.M., Ryabchenko K.A., Orrico E.G., Gibb B.E. (2005). Self-mutilation and symptoms of depression, anxiety, and borderline personality disorder. Suicide Life-Threat. Behav..

[53] Muehlenkamp J.J., Gutierrez P.M. (2007). Risk for suicide attempts among adolescents who engage in non-suicidal self-injury. Arch. Suicide Res..

[54] Gardner D.L., Cowdry R.W. (1985). Suicidal and parasuicidal behavior in borderline personality disorder. Psychiatr. Clin. N. Am..

[55] Favazza A.R., Conterio K. (1989). Habitual female self-mutilators. Acta Psychiatr. Scand..

[56] Ross S., Heath N. (2002). A study of the frequency of self-mutilation in a community sample of adolescents. J. Youth Adolesc..

[57] Klonsky E.D., Oltmanns T.F., Turkheimer E. (2003). Deliberate self-harm in a nonclinical population: Prevalence and psychological correlates. Am. J. Psychiatry.

[58] Nock M.K., Prinstein M.J. (2004). A functional approach to the assessment of selfmutilative behavior. J. Consult. Clin. Psychol..

[59] Nock M.K., Prinstein M.J. (2005). Contextual Features and Behavioral Functions of Self-Mutilation among Adolescents. J. Abnorm. Psychol..

[60] Guerry J.D., Prinstein M.J. (2009). Longitudinal prediction of adolescent nonsuicidal self-injury: Examination of a cognitive vulnerability-stress model. J. Clin. Child Adolesc. Psychol..

[61] Nock M.K., Mendes W.B. (2008). Physiological arousal, distress tolerance, and social problem-solving deficits among adolescent self-injurers. J. Consult. Clin. Psychol..

[62] Nock M.K., Favazza A., Nock M.K. (2009). Nonsuicidal self-injury: Definition and classification. Understanding Nonsuicidal Selfinjury: Origins, Assessment, and Treatment.

[63] Shields C.G., Franks P., Harp J.J., Campbell T.L., McDaniel S.H. (1994). Family Emotional Involvement and Criticism Scale (FEICS): II. Reliability and validity studies. Fam. Syst. Med..

[64] Andrews B., Qian M., Valentine J.D. (2002). Predicting depressive symptoms with a new measure of shame: The Experience of Shame Scale. Br. J. Clin. Psychol..

[65] Lovibond S.H., Lovibond P.F. (1995). The Structure of Negative Emotional States; Comparison of the Depression Anxiety Stress Scales (DASS) with the Beck Depression and Anxiety Inventories. Behav. Res. Ther..

[66] Rotolone C., Martin G. (2012). Giving up self-injury: A comparison of everyday social and personal resources in past versus current self-injurers. Arch. Suicide Res..

[67] Gratz K.L. (2001). Measurement of deliberate self-harm: Preliminary data on the deliberate self-harm inventory. J. Psychopathol. Behav. Assess..

[68] Yates T.M., Tracy A.J., Luthar S.S. (2008). Nonsuicidal self-injury among “privileged” youths: Longitudinal and cross-sectional approaches to developmental process. J. Consult. Clin. Psychol..

[69] Sornberger M.J., Heath N.L., Toste J.R., McLouth R. (2012). Nonsuicidal Self-Injury and Gender: Patterns of Prevalence, Methods, and Locations among Adolescents. Suicide Life-Threat. Behav..

[70] Suyemoto K.L. (1998). The functions of self-mutilation. Clin. Psychol. Rev..

[71] Bachtelle S.E., Pepper C.M. (2015). The Physical Results of Nonsuicidal Self-Injury: The Meaning behind the Scars. J. Nerv. Ment. Dis..

[72] Duggan J., Heath N., Hu T. (2015). Non-suicidal self-injury maintenance and cessation among adolescents: A one-year longitudinal investigation of the role of objectified body consciousness, depression and emotion dysregulation. Child Adolesc. Psychiatry Ment. Health.

[73] Brodsky B.S., Cloitre M., Dulit R.A. (1995). Relationship of dissociation to self-mutilation and childhood abuse in borderline personality disorder. Am. J. Psychiatry.

[74] Simeon D., Stanley B., Frances A., Mann J.J., Winchel R., Stanley M. (1992). Self-mutilation in personality disorders: Psychological and biological correlates. Am. J. Psychiatry.

